# The Association of obesity with vascular complications after liver transplantation

**DOI:** 10.1186/s12876-019-0954-8

**Published:** 2019-03-07

**Authors:** Yi Shi, Bingsong Huang, Ronghai Deng, Yi Ma

**Affiliations:** Department of Organ Transplantation, First Affiliated Hospital of Sun Yat-sen University, No. 58 Zhongshan 2nd Road, Guangzhou, 510080 China

**Keywords:** Liver transplantation, Vascular complications, Obese, Body mass index, Meta-analysis

## Abstract

**Background:**

Because of the growing number of obese patients undergoing liver transplantation (LT), it is important to investigate the impact of obesity on post-transplant outcomes. Vascular complications are rare, but serious causes of morbidity and mortality after LT. It is not known if pre-transplant obesity is associated with an increased incidence of post-LT vascular complications.

**Methods:**

Medline, Embase, and Cochrane Library databases were searched in September 2017. The primary outcome was the impact of obesity on the vascular complication rate in adult LT recipients. Survival and biliary complications rates were also analyzed. Risk ratios (RRs) and 95% confidence intervals (CIs) were calculated to compare pooled data between groups with a body mass index (BMI) ≥ 30 kg/m^2^ and < 30 kg/m^2^.

**Results:**

Six retrospective cohort studies with a total of 987 patients with a BMI ≥ 30 kg/m^2^ (high BMI group) and 2911 patients with a BMI < 3 0 kg/m^2^ (control group) were included in the analysis. All studies had Newcastle-Ottawa Scale scores ≥4. The vascular complication rates were similar between the high BMI group and control group (RR = 1.13, 95% CI: 0.87–1.47, *P* = 0.27), as were the patient survival, graft survival, and biliary complication rates. In subgroup analysis, there was no difference in the vascular complication rates between BMI ≥ 35 vs. BMI < 25 kg/m^2^; BMI 30–35 vs. BMI 18–25 kg/m^2^; BMI ≥ 30 vs. BMI 18–25 kg/m^2^; and BMI ≥ 35 vs. BMI < 35 kg/m^2^. No difference was found in subgroup analysis when BMI was adjusted for ascites. However, recipients whose primary disease was alcoholic liver disease, those with a BMI ≥ 30 kg/m^2^ had higher incidence of vascular complications than those with a BMI < 30 kg/m^2^ (RR = 1.55, 95% CI: 1.07–2.25, *P* = 0.02) .

**Conclusions:**

BMI does not affect incidence of vascular complications after LT. High pre-transplant BMI is not a risk factor for patient survival and biliary complications after LT.

**Electronic supplementary material:**

The online version of this article (10.1186/s12876-019-0954-8) contains supplementary material, which is available to authorized users.

## Introduction

The World Health Organization (WHO) reported that 13.2% of global adults were obese in 2016 [1]. Obesity has become a global epidemic that contributes to a number of comorbidities and metabolic disorders [2, 3]. Furthermore, the number of obese patients undergoing liver transplantation (LT) is rising rapidly in the United States [4–6], and in the rest of the world. The proportion of obese patients in LT recipients was 33% between 2002 and 2011 [7], as compared to 20% between 1988 and 1996 [8]. Moreover, it was recently reported that severely and morbidly obese patients (body mass index [BMI] > 35 kg/m^2^) patients account for 7–12% of LT recipients [8, 9].

As the number of patients on the transplant waiting list is increasing, and with the lack of sufficient donors to meet this demand, it is important to predict the prognosis of every candidate to help decide the allocation of donor livers. Vascular complications after LT are associated with a high incidence of graft loss and mortality because of the impact on blood flow [10]. Bleeding, stenosis, thrombosis, and an aneurysm can occur at any vascular anastomoses, with a reported rate of 8–15% [11]. Causes of vascular complications include technical problems in anastomosis and allograft anatomy [12]. La Mattina et al. [13] reported that obesity may be a risk factor for vascular complications after LT. The authors reviewed 813 LT recipients, and divided them into 5 groups according to BMI. There was a significantly higher rate of deep venous thrombosis in patients with a BMI > 40 kg/m^2^ compared to those with a normal BMI. Other studies also reported that obese LT recipients had a trend toward a higher risk of vascular complications after transplantation, though differences between groups were not statistically significant [14, 15]. On the other hand, a retrospective cohort study including 1325 LT recipients showed that obese patients had a slightly decreased rate of vascular complications, but again the difference between groups was not significant [16]. Spengler et al. [17] reviewed studies that reported morbidity after LT in obese versus non-obese patients, and found postoperative infections and respiratory failure occurred more frequently in obese recipients. A meta-analysis by Saab et al. [18] including 13 studies found that obesity negatively impacted survival in LT recipients, but vascular complications were not studied. As such, it is not known if obesity affects the rate of vascular complications after LT.

The objective of this study was to conduct a meta-analysis to determine the impact of BMI on vascular complications in LT recipients.

## Materials and methods

### Data sources and literature searches

PubMed/Medline, Embase, and Cochrane Library databases were searched in September 2017 using the terms “complication or complications” AND “body mass index or overweight or obesity or obese” AND “liver transplant or liver transplantation”. Eligible publications were limited to those written in English, and to those reporting results from human subjects. Review and meta-analysis articles were excluded after limit filtering. A manual search of the references of relevant publications was also performed.

### Study inclusion and exclusion criteria

Studies reporting outcomes of LT in different preoperative BMI groups were included. Exclusion criteria were: 1) Overlapping cohort studies from the same institution (to avoid duplication); 2) Studies in which post-transplantation vascular complications were not reported in patients with a BMI ≥ 30 kg/m^2^ and those with a BMI < 30 kg/m^2^; 3) Studies of pediatric patients.

### Quality assessment and data extraction

The methodological quality of observational studies was determined using the Newcastle-Ottawa Scale. ‘Selection’, ‘comparability,’ and ‘outcome’ are the 3 categories included in the Newcastle-Ottawa Scale for cohort studies. Publications were assessed and data were extracted by 2 independent investigators, with disagreements resolved through discussion and consensus.

The primary outcome of the meta-analysis was the incidence of vascular complications following LT, which were comprised of stenosis, thrombosis, and pseudoaneurysm. Secondary outcomes were biliary complications, and patient and graft survival rates. Given that BMI is a universal measure to determine obesity, a BMI ≥ 30 kg/m^2^ was chosen as the criterion of obesity. Results of patients with a BMI ≥ 30 kg/m^2^ were compared with those of patients with a BMI < 30 kg/m^2^.

### Data synthesis and analysis

Risk ratios (RRs) were used to evaluate the event rates, and the results were reported with 95% confidence intervals (CIs). *P* values < 0.05 were considered to indicate a significant difference between the 2 groups. Heterogeneity of the included studies was evaluated by the χ^2^ and Ι^2^ statistical tests. Heterogeneity was considered significant when χ^2^ value of P was < 0.05, or Ι^2^ was > 50%: in this case a random-effects model of analysis was used. When significant heterogeneity was not identified, a fixed-effects model of analysis was used. Publication bias was assessed by a funnel plot. All statistical analysis was performed using Review Manager software (RevMan Version 5.3).

## Results

### Search results and included studies

**Fig. 1:**
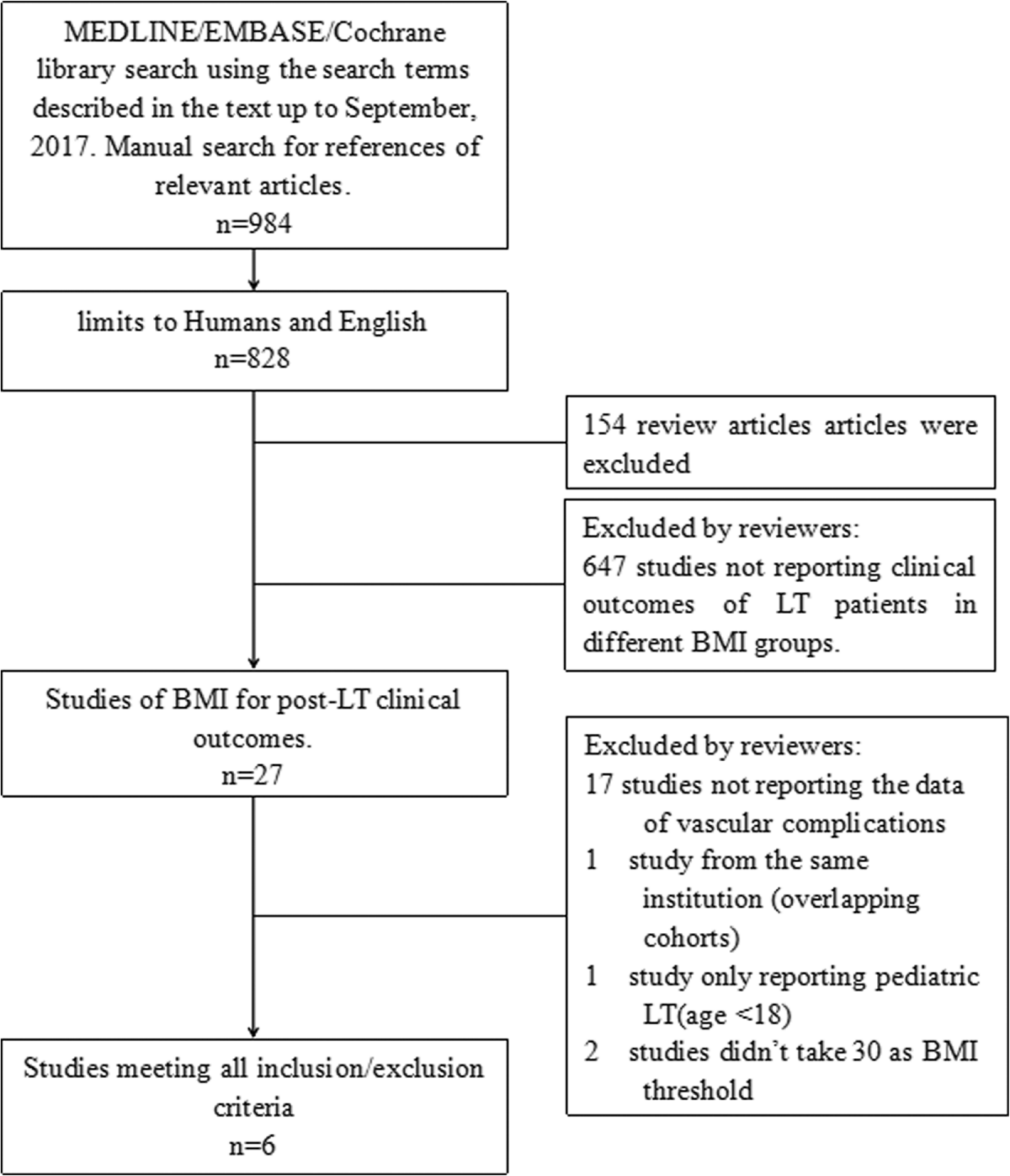
Search algorithm and study selection

A total of 984 articles were identified in the database searches (Fig. 1). After excluding animal studies and non-English language reports, 828 studies remained. Of these, 154 review articles and 647 studies not reporting pre-transplant BMI were excluded. The remaining 27 publications underwent extensive review. Seventeen studies lacking data of vascular complications and 1 study [19] of pediatric patients were excluded. There were 2 studies with overlapping cohorts from the same institution [14, 20], and only the latest report [14] was included. Two studies did not use 30 kg/m^2^ as the BMI threshold. We requested the primary data from the corresponding authors, but did not receive replies so these studies were excluded from the primary analysis. Finally, 6 studies meeting all of the criteria were included in the meta-analysis. However, the 2 studies in which the BMI threshold was not 30 kg/m^2^ were included in the subgroup analysis.

The characteristics of the 8 studies are summarized in Table 1. No evidence of publication bias among the included studies was found by funnel plot examination. In the 6 included studies, there were 2911 LT recipients with a preoperative BMI < 30 kg/m^2^ and 987 with a preoperative BMI ≥ 30 kg/m^2^. Three of the studies were from European countries, 2 were from the United States, and 1 was from Canada. When assessed with the Newcastle-Ottawa Scale, all studies obtained a score ≥ 4 stars, with a highest score of 6 (Table 2).

**Table 1:** Characteristics of the included studies

Reference	Region	Sample Size		Periods	Recipient Age		MELD Score	Male (%)	Most common etiology
		BMI ≥ 30	BMI < 30		BMI ≥ 30	BMI < 30			
Nair (2001)^a^ [15]	America	21^Φ^	64^b^	1994-1996	46.7 ± 10.5^Φ^	46.0 ± 12.6^b^	/	82.4	HCV
Fujikawa (2006) [21]	America	167	533	1990–2005	51.0 ±?	49.5 ±?	18.5 ±?	61.3	/
Lamattina (2012) [13]	America	306	482	1997–2008	53.4 ± 8.8	52.6 ± 9.9	19.8 ± 8.8	63.1	Alcohol
Hakeem (2013) [16]	The UK	218	1107	1994–2009	50.9 ± 8.5	47.4 ± 13.0	19.7 ± 10.7	56.9	cholestasis
Tanaka (2013) [22]	Canada	149	358	2000–2006	52.9 ± 8.6	51.4 ± 10.5	16.2 ± 8.0	69.0	HCV
Gunay (2013) [23]	Europe	74	296	2004–2012	52.4 ± 8.4	50.4 ± 11.3	16.7 ± 6.6	76.8	HBV
Triguero (2015)^a^ [24]	Europe	11(BMI > 35)	36(BMI 20–25)	2007–2013	53 ±?(45–64)	54.5 ±?	15 ±?	78.7	Alcohol
Molina (2016) [14]	Europe	73	135	2008–2014	54.8 ± 7.7	52.8 ± 10.3	18.2 ± 5.0	84.1	Alcohol

^a^These studies were not included because of inconsistent grouping with others. Φ: BMI > 31.1 for men and 32.3 for women

^b^BMI < 27.8 for men and < 27.3 for women; MELD: model for end-stage liver disease; HBV: hepatic B virus; HCV: hepatic C virus

**Table 2:** Quality assessment using the Newcastle-Ottawa scale

Studies	The Newcastle-Ottawa Scale								
	Selection				Comparability	Outcome			Total score
	Representativeness of the exposed cohort (maximum:★)	Selection of the non exposed cohort (maximum:★)	Ascertainment of exposure (maximum:★)	Demonstration that outcome of interest was not present at start of study (maximum:★)	Comparability of cohorts on the basis of the design or analysis (maximum:★★)	Assessment of outcome (maximum:★)	Was follow-up long enough for outcomes to occur (maximum:★)	Adequacy of follow up of cohorts (maximum:★)	
Nair (2001) [15]	★	★	★	–	–	★	★	–	★★★★★
Fujikawa (2006) [21]	★	★	★	–	–	–	★	–	★★★★
Lamattina (2012) [13]	★	★	★	–	–	–	★	–	★★★★
Hakeem (2013) [16]	★	★	★	–	–	★	★	–	★★★★★
Tanaka (2013) [22]	★	★	★	–	–	★	★	–	★★★★★
Gunay (2013) [23]	–	★	★	★	–	★	★	★	★★★★★★
Triguero (2015) [24]	★	★	★	–	–	★	★	–	★★★★★
Molina (2016) [14]	★	★	★	–	–	★	★	–	★★★★★

### Vascular complications

Six studies reported the incidence of vascular complications in patients with a BMI ≥ 30 kg/m^2^ and BMI < 30 kg/m^2^ [13, 14, 16, 21–23]. Additionally, we also included the 2 studies in which the BMI threshold was not 30 kg/m^2^ in subgroup analysis. The study by Triguero et al. included 2 groups: BMI ≥ 35 kg/m^2^ and BMI 18–25 kg/m^2^ [24]. The study by Nair et al. divided women into those with a BMI < 27.3 kg/m^2^, BMI 27.3–32.3 kg/m^2^, and BMI > 32.3 kg/m^2^, and men into those with a BMI < 27.8 kg/m^2^, BMI 27.8–31.1 kg/m^2^, and BMI > 31.1 kg/m^2^ [15]. We excluded the intermediate classes, and used the remaining 2 groups as the control and high BMI group.

**Fig. 2:**
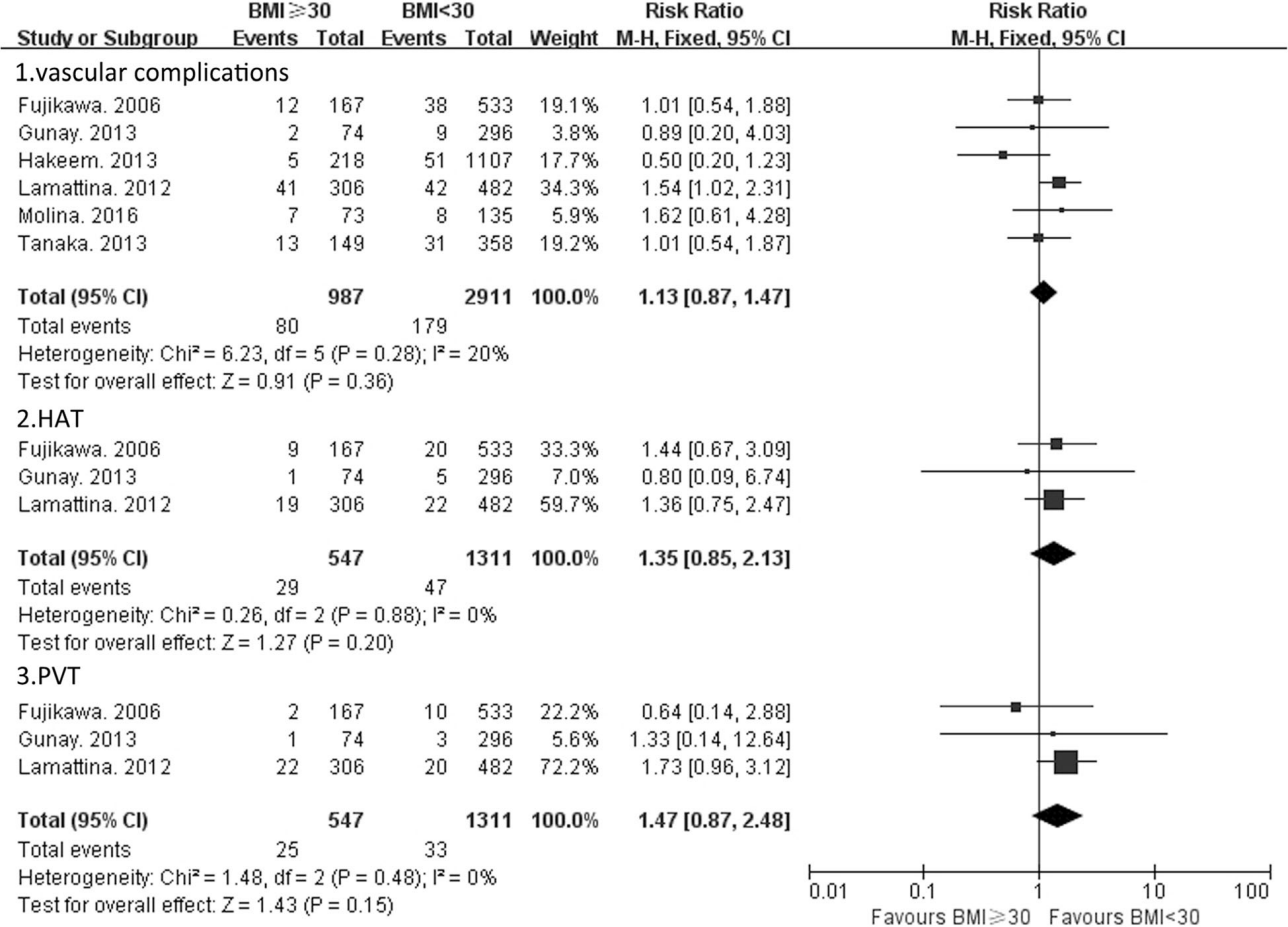
Vascular complication rates for LT recipients with a BMI ≥ 30 kg/m^2^ versus a BMI < 30 kg/m^2^

None of the studies reported a significant difference in the vascular complication rate between BMI groups. The overall rate of vascular complications among the 6 studies was 12.50% (range: 2.29–40%) for BMI ≥ 30 kg/m^2^ (*n* = 987), and 6.15% (range: 3.04–8.71%) for BMI < 30 kg/m^2^ (*n* = 2911). There was no significant heterogeneity among the 6 studies (χ^2^ = 6.23, *P* = 0.28; Ι^2^ = 20%). Thus, a fixed-effects model of analysis was used. The pooled result of the 6 studies showed that the RR for vascular complications was 1.13 (95% CI: 0.87–1.47) for BMI ≥ 30 kg/m^2^ as compared to a BMI < 30 kg/m^2^ (*P* = 0.36) (Fig. 2). If the other 2 studies in which the BMI threshold was not 30 kg/m^2^ were also included, the difference in the vascular complications rate between the high BMI group and control group was still not significant (RR = 1.13, 95% CI: 0.88–1.46, *P* = 0.34).

Considering that the study by Nair et al. whose patients underwent LT between 1994 and 1996 was relatively old, we excluded it and analyzed the remaining 7 studies. No significant difference in the vascular complication rate was noted (RR = 1.11, 95% CI: 0.86–1.44, *P* = 0.41).There were 3 studies that reported the incidence of hepatic arterial thrombosis (HAT) and portal venous thrombosis (PVT) [13, 21, 23]. No significant difference in HAT incidence was found between patients with a BMI ≥ 30 kg/m^2^ (*n* = 547) and BMI < 30 kg/m^2^ (*n* = 1311) (RR = 1.35, 95% CI: 0.85–2.13, *P* = 0.20). PVT incidence was also similar between patients with a BMI ≥ 30 kg/m^2^ (n = 547) and BMI < 30 kg/m^2^ (n = 1311) (RR = 1.47, 95% CI: 0.87–2.48, *P* = 0.15).

### BMI threshold

Data from 4 studies [13, 14, 16, 22] were available to compare the vascular complication rates between LT recipients with a BMI ≥ 35 kg/m^2^ (*n* = 246) and a BMI < 25 kg/m^2^ (*n* = 1149). No difference was found in incidence of vascular complications between these groups (RR = 1.35, 95% CI: 0.81–2.23, *P* = 0.25) (Additional file 1). Likewise, there was no difference in the incidence of vascular complications between recipients with a BMI of 30–35 kg/m^2^ (*n* = 500) and 18–25 kg/m^2^ (*n* = 1169) (RR = 1.22, 95% CI: 0.80–1.86, *P* = 0.36) (Additional file 2) [13, 14, 16, 22]. Based on data from 4 studies [13, 16, 22, 23], there was no difference in the incidence of vascular complications incidence between patients with a BMI ≥ 30 kg/m^2^ (*n* = 747) and BMI 18–25 kg/m^2^ (n = 1169) (RR = 1.11, 95% CI: 0.55–2.22, *P* = 0.77) (Additional file 3) Subgroup analysis using a BMI threshold of 35 kg/m^2^ with data from 4 studies [13, 14, 16, 22] found no difference in the vascular complications incidence between recipients with a BMI ≥ 35 kg/m^2^ (*n* = 246) and a BMI < 35 kg/m^2^ (*n* = 2568) (Additional file 4) (RR = 1.22, 95% CI: 0.80–1.85, *P* = 0.36).

### Ascites

The pooled analysis of 4 studies [13, 14, 16, 22] which adjusted for the presence of ascites showed no difference in survival between patients with a BMI ≥ 30 kg/m^2^ (*n* = 671) and a BMI < 30 kg/m^2^ (*n* = 2020) (RR = 1.21, 95% CI: 0.87–1.68, *P* = 0.27) (Additional file 5). Further analysis of pooled studies which corrected BMI for ascites revealed no difference in the rate of vascular complications (BMI 30–35 vs. BMI 18–25 kg/m^2^: RR = 1.07, 95% CI: 0.66–1.74, *P* = 0.77; BMI ≥ 35 vs. BMI < 35 kg/m^2^: RR = 1.22, 95% CI: 0.80–1.85, P = 0.36). No difference was also found in the pooled analysis of studies that did not control for ascites (RR = 1.01, 95% CI: 0.65–1.56, *P* = 0.97) [21, 22]. In the analysis, there were 337 patients with a BMI ≥ 30 kg/m^2^ and 955 patients with a BMI < 30 kg/m^2^.

### Etiology

Subgroup analysis was performed according to the etiology of liver disease. Alcoholic liver disease was the most common indication for LT in 2 studies [13, 14]. Pooled analysis of the 2 studies showed that the patients with a BMI ≥ 30 kg/m^2^ (*n* = 379) had a significant higher incidence of vascular complications than patients with a BMI < 30 kg/m^2^ (*n* = 617) (RR = 1.55, 95% CI: 1.07–2.25, *P* = 0.02). In the analysis of pooled data from 2 studies where viral hepatitis was the most common indication for LT [22, 23], there was no significant difference in vascular complications between patients with a BMI ≥ 30 kg/m^2^ (*n* = 223) and a BMI < 30 kg/m^2^ (*n* = 654) (RR = 0.99, 95% CI: 0.56–1.75, *P* = 0.97).

### Patient survival

**Fig. 3:**
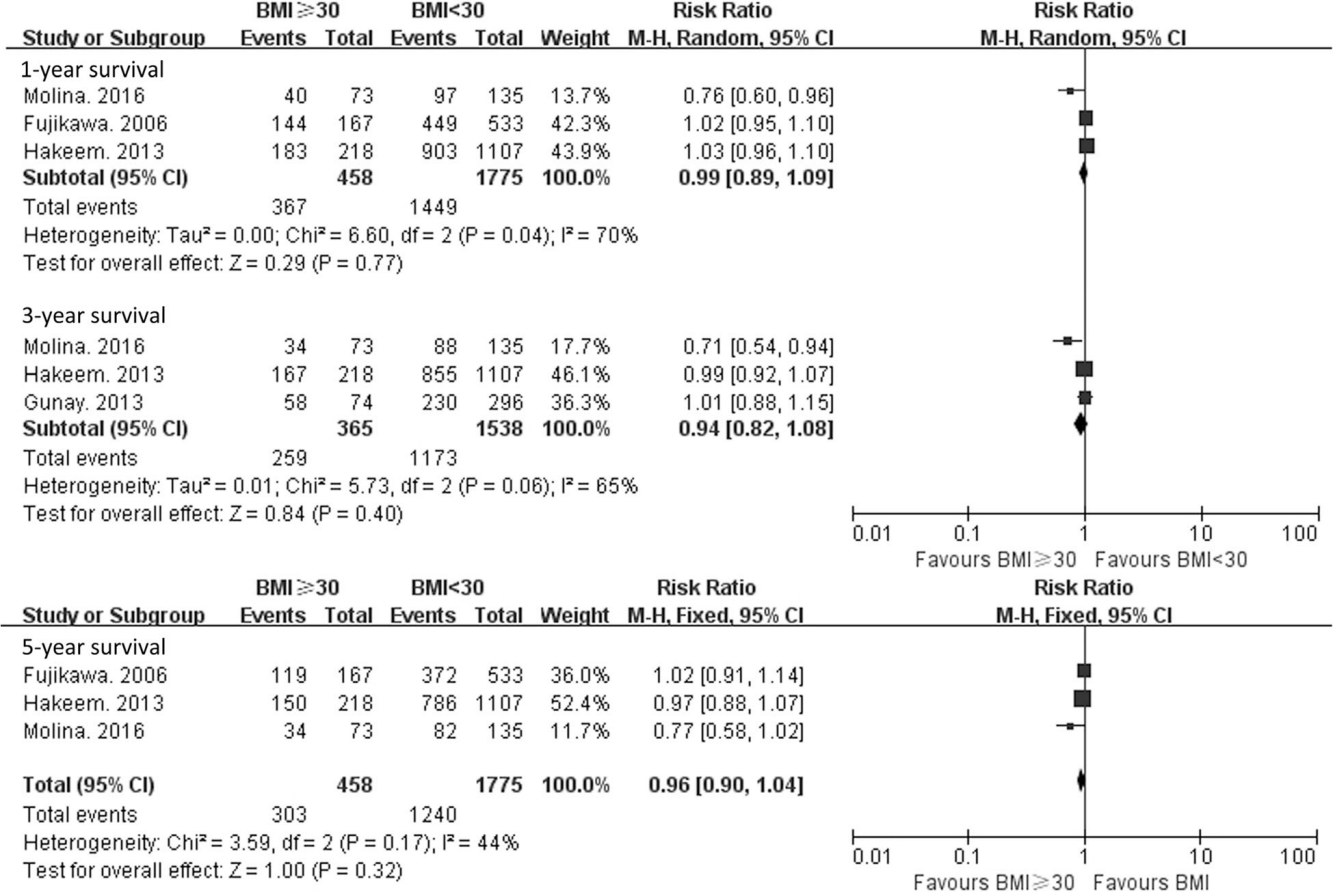
Survival of LT recipients with a BMI ≥ 30 kg/m^2^ versus a BMI < 30 kg/m^2^

We evaluate patient survival at 1, 3, and 5 years after transplantation. Three of the 6 included publications reported 1- and 5-year survival rates [14, 16, 21], and 3 reported 3-year patient survival rates [14, 16, 23]. There was significant heterogeneity among the 3 studies that reported 1-year survival rate (χ^2^ = 6.60, *P* = 0.04; Ι^2^ = 7 0%) (Fig. 3). Similarly, significant heterogeneity was observed among the 3 studies reporting 3-year patient survival rates (χ^2^ = 5.73, *P* = 0.06; Ι^2^ = 65%). The heterogeneity was not significant in the 3 studies that reported 5-year patient survival rates (χ^2^ = 3.59, *P* = 0.17, Ι^2^ = 44%). Therefore, a random-effects model was used to pool the overall results for 1- and 3-year survival rates, and a fixed-effects model was used for analysis of 5-year survival rates. For patients with a BMI ≥ 30 kg/m^2^, the RR for 1-, 3- and 5- survival was 0.99 (95% CI: 0.89–1.09, *P* = 0.77), 0.94 (95% CI: 0.82–1.08, *P* = 0.40), and 0.96 (95% CI: 0.90–1.04, *P* = 0.32). There was no significant difference in the 1-, 3-, and 5-year survival rate between the 2 groups.

### Graft survival

**Fig. 4:**
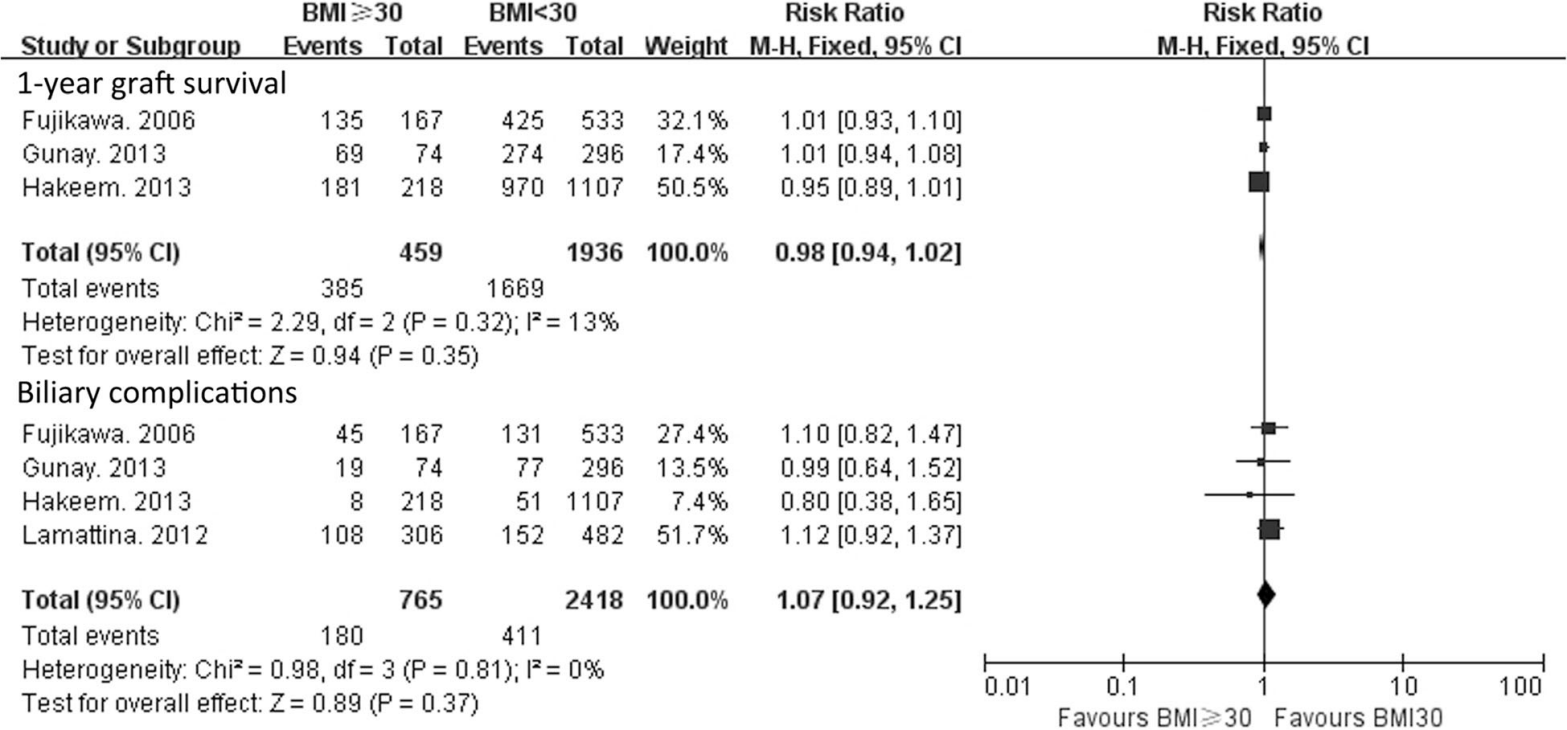
1-year graft survival and biliary complication rates for LT recipients with a BMI ≥ 30 kg/m^2^ versus a BMI < 30 kg/m^2^

Three studies reported 1-year graft survival rate [16, 21, 23]. The heterogeneity among these studies was not significant (χ^2^ = 2.29, P = 0.32; Ι^2^ = 13%); thus, a fixed-effects model was used. There was no significant difference in 1-year graft survival between patients with a BMI ≥ 30 kg/m^2^ (*n* = 459) and a BMI < 30 kg/m^2^ (*n* = 1936) (RR = 0.98, 95% CI: 0.94–1.02, *P* = 0.35) (Fig. 4).

### Biliary complications

We further evaluate the rate of biliary complications. Four studies including 765 patients with a BMI ≥ 30 kg/m^2^ and 2418 patients with a BMI < 30 kg/m^2^ were included in the analysis of biliary complications [13, 16, 21, 23]. No significant difference between the 2 groups was noted (RR = 1.07, 95% CI: 0.92–1.25, *P* = 0.37) (Fig. 4).

## Discussion

As the number of obese persons rises globally, the trend that obese patients make up a growing proportion of LT recipients is unavoidable. There is no certain conclusion whether obesity impacts the incidence of post-transplant vascular complications in LT patients. This is the first meta-analysis to examine the association of obesity with vascular complications after LT. The results of our study indicate that obesity does not increase the rate of vascular complications in LT recipients, nor does obesity increase the rate of HAT or PVT. However, in pooled analysis of 2 studies where the indication for LT was alcoholic cirrhosis, obese patients had significantly higher incidence of vascular complications.

Subgroup analysis performed according to different BMI thresholds and adjustment of BMI for ascites also failed to identify any significant difference between patients with a BMI ≥ 30 kg/m^2^ and a BMI < 30 kg/m^2^. Similarly, a review of the United NetWork of Organ Sharing (UNOS) database (1987–2001) that included 9701 pediatric LT recipients showed that the vascular thrombosis rate was not higher in obese versus non-obese recipients [19]. However, a retrospective analysis of renal transplantation including 1095 patients found the risk of post-transplant vascular complications within 1 year was greater when grafts were transplanted with multiple arteries in obese recipients compared to their non-obese counterparts [25]. The hypercoagulable and proinflammatory state associated with obesity predisposing to both arterial and venous thrombosis was proposed to explain the result.

La Mattina et al. [13] thought that technical difficulties and a high rate of metabolic syndrome in obese LT recipients contribute to a higher rate of post-transplant vascular complications. However, the reasons for this are not clear and probably multi-factorial. We propose some reasons that may explain the lack of association between obesity and post-transplant outcomes in LT recipients. It is possible that obesity, in fact, does not impact the occurrence of vascular complications. In addition, there is strict screening of patients prior to LT, and optimization of risk factors. Another explanation is that BMI does not accurately represent the degree of obesity. The distribution of fat deposition is not accounted for in BMI. Study has shown that visceral adiposity, but not peripheral adiposity, is associated with increased mortality after LT [26].

In the analysis of 3 studies that reported the patient survival, we found no difference in the 1-, 3-, and 5-year survival between patients with a BMI ≥ 30 kg/m^2^ and BMI < 30 kg/m^2^. Some recent publications also reported no significant association between obesity and patient survival after LT [7, 27]. Biliary complications are intrinsically linked with hepatic arterial pathology [28–30]. Our analysis showed no difference in the rate of biliary complications between obese versus non-obese LT recipients.

There are some limitations to this study. First, there were no randomized controlled trials published; all of the included studies were retrospective cohort studies. The quality of the studies was not satisfactory because none of them adjusted for confounders. As such, heterogeneity between studies was unavoidable. Second, all of the included studies were single-center studies. In addition, none of the studies described how complications were diagnosed.

## Conclusions

The current analysis indicates that obesity does not increase the rate of vascular complications in LT recipients. Patient survival at 1-, 3-, and 5-years and the biliary complication rate were also similar between obese and non-obese patients. Overall, a high BMI should not be a contraindication for LT. However, obese recipients with alcoholic liver disease and metabolic syndrome should be monitored closely for vascular complications.

## Additional files

**Additional file 1:** Vascular complications rate for LT recipients with BMI≥35 versus BMI<25. (TIF 1388 kb)

**Additional file 2:** Vascular complications rate for LT recipients with BMI of 30-35 versus 18-25. (TIF 1448 kb)

**Additional file 3:** Vascular complications rate for LT recipients with BMI≥30 versus 18-25. (TIF 1445 kb)

**Additional file 4:** Vascular complications rate for LT recipients with BMI≥35 versus <35. (TIF 1386 kb)

**Additional file 5:** Vascular complications rate for LT recipients with BMI≥30 versus <30 in studies adjusted BMI for ascites. (TIF 1386 kb)

## Abbreviations

BMI: Body mass index
CI: Confidence interval
HAT: Hepatic arterial thrombosis
HBV: Hepatic B virus
HCV: Hepatic C virus
LT: Liver transplantation
MELD: Model for end-stage liver disease
PVT: Portal venous thrombosis
RevMan: Review Manager
RR: Risk ratio
UNOS: United NetWork of Organ Sharing
WHO: the World Health Organization
