# Supplementary figures and images for: The Association of obesity with vascular complications after liver transplantation
Source: BMC Gastroenterol. 2019 Mar 7;19:39. doi: 10.1186/s12876-019-0954-8 (PMC6407261; doi:10.1186/s12876-019-0954-8)

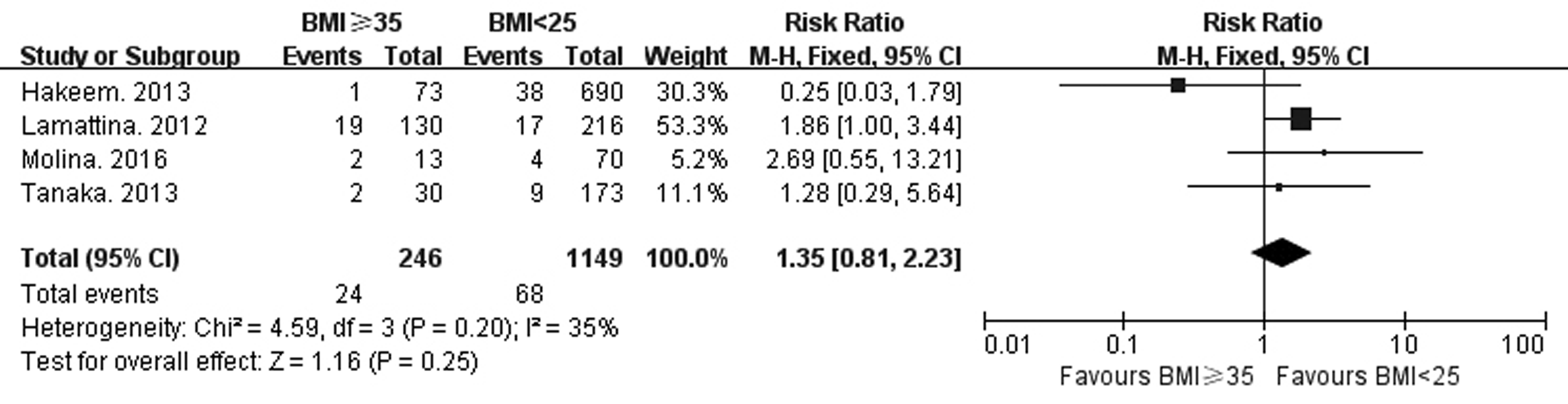

Supplement: Supplementary file 1 — Additional file 1: Vascular complications rate for LT recipients with BMI≥35 versus BMI<25. (TIF 1388 kb) [file 12876_2019_954_MOESM1_ESM.tif]

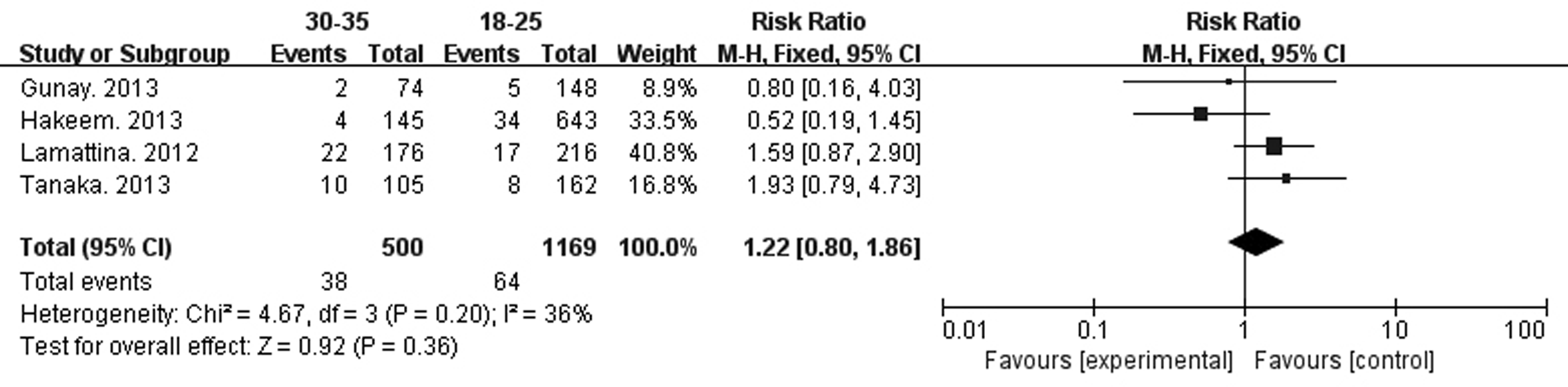

Supplement: Supplementary file 2 — Additional file 2: Vascular complications rate for LT recipients with BMI of 30-35 versus 18-25. (TIF 1448 kb) [file 12876_2019_954_MOESM2_ESM.tif]

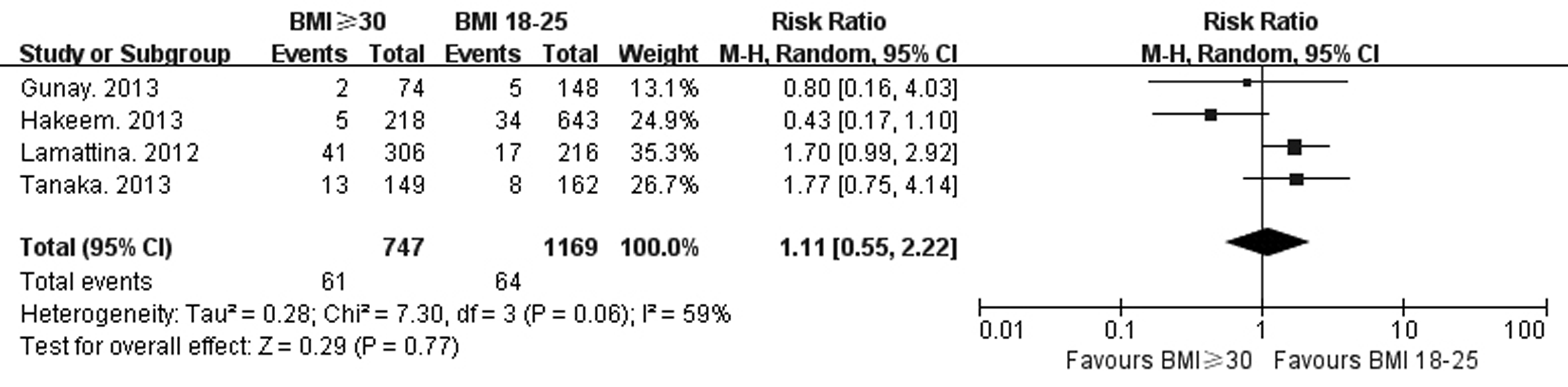

Supplement: Supplementary file 3 — Additional file 3: Vascular complications rate for LT recipients with BMI≥30 versus 18-25. (TIF 1445 kb) [file 12876_2019_954_MOESM3_ESM.tif]

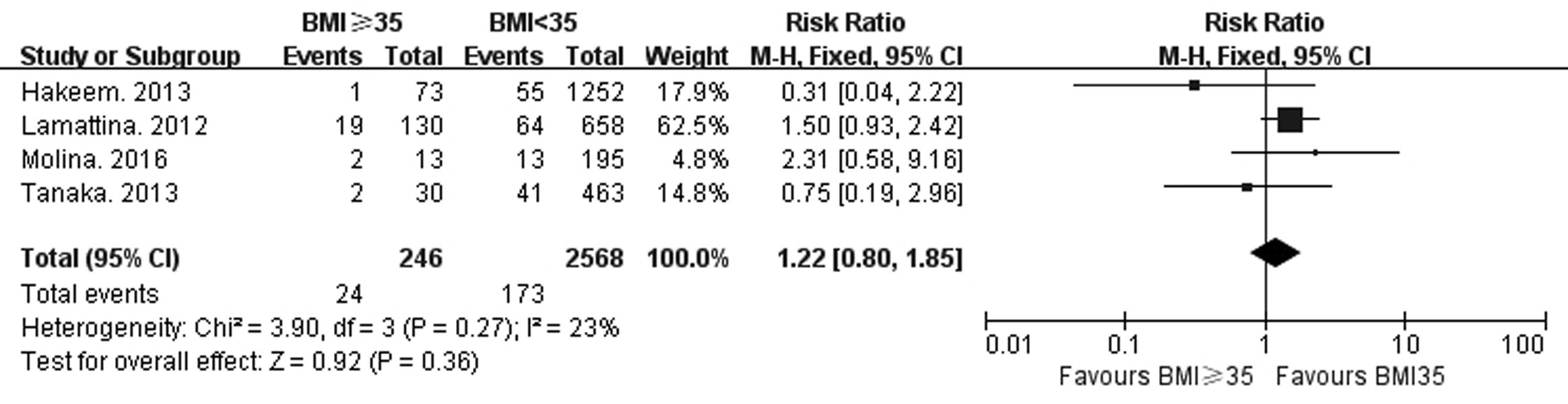

Supplement: Supplementary file 4 — Additional file 4: Vascular complications rate for LT recipients with BMI≥35 versus <35. (TIF 1386 kb) [file 12876_2019_954_MOESM4_ESM.tif]

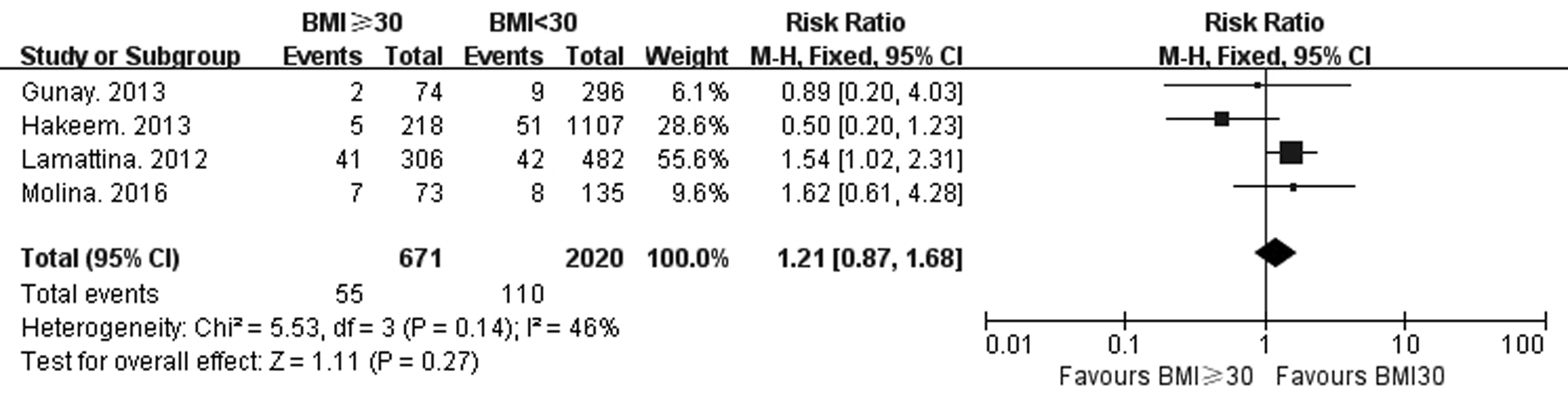

Supplement: Supplementary file 5 — Additional file 5: Vascular complications rate for LT recipients with BMI≥30 versus <30 in studies adjusted BMI for ascites. (TIF 1386 kb) [file 12876_2019_954_MOESM5_ESM.tif]
